# HIV, opioid use, and alterations to the gut microbiome: elucidating independent and synergistic effects

**DOI:** 10.3389/fimmu.2023.1156862

**Published:** 2023-04-24

**Authors:** Sanjana Satish, Yaa Abu, Daniel Gomez, Rajib Kumar Dutta, Sabita Roy

**Affiliations:** ^1^ Department of Medical Education, University of Miami Miller School of Medicine, Miami, FL, United States; ^2^ Department of Surgery, University of Miami Miller School of Medicine, Miami, FL, United States

**Keywords:** HIV, opioid use disorder, medication assisted treatment, gut microbiome, fecal microbial transplantation, probiotics, antiretroviral therapy

## Abstract

**Background:**

The microbiome is essential to immune development, defense against pathogens, and modulation of inflammation. Microbial dysbiosis has been reported in various diseases including human immunodeficiency virus (HIV) and opioid use disorder (OUD). Notably, people living with HIV (PLWH) have been reported to both have higher rates of OUD and use opioids at higher rates than the general public. Thus, studying gut microbial alterations in people living with HIV and with OUD could elucidate mechanisms pertaining to how these conditions both shape and are shaped by the microbiome. However, to date few studies have investigated how HIV and OUD in combination impact the microbiome.

**Aim of review:**

Here, we review previous studies outlining interactions between HIV, opioid use, and microbial dysbiosis and describe attempts to treat this dysbiosis with fecal microbial transplantation, probiotics, and dietary changes.

**Key scientific concepts of review:**

While the limited number of studies prevent overgeneralizations; accumulating data suggest that HIV and opioid use together induce distinct alterations in the gut microbiome. Among the three existing preclinical studies of HIV and opioid use, two studies reported a decrease in *Lachnospiraceae* and *Ruminococcaceae*, and one study reported a decrease in *Muribaculaceae* in the combined HIV and opioid group relative to HIV-alone, opioid-alone, or control groups. These bacteria are known to modulate immune function, decrease colonic inflammation, and maintain gut epithelial barrier integrity in healthy individuals. Accordingly, modulation of the gut microbiome to restore gut homeostasis may be attempted to improve both conditions. While mixed results exist regarding treating dysbiosis with microbial restoration in PLWH or in those with opioid dependency, larger well-defined studies that can improve microbial engraftment in hosts hold much promise and should still be explored.

## 1 Introduction

The microbiota consists of the many microorganisms, including viruses, bacteria, and fungi, that exist on and within the human body (1). Residing in the skin, lungs, oral cavity, vagina, and gastrointestinal (GI) tract, there are about 100 trillion currently known microorganisms (1). Together, these microorganisms work in a symbiotic relationship with the body and play a crucial role in overall host homeostasis, particularly through development and maintenance of immune function, and modulation of host nutrition and energy metabolism (2). Additionally, microbial metabolites such as tryptophan, polyamines, and amino acids influence human behavior and physiology (3). Gamma-aminobutyric acid (GABA), for example, is a known regulator of the central and enteric nervous systems and histamine has been associated with regulation of intestinal immune cells (4, 5). Short chain fatty acids (SCFAs) produced by GI microbiota ensure gut homeostasis through immune regulation, nutrient absorption, pH regulation, and mucus secretion (4). In addition to the GI tract, whose microorganisms and their contributions to the human microbiome are the most well-known, many other body organs and systems have now been found to harbor a distinct microbiome (6, 7).

Even among different body sites, it is known that microbial composition is dependent on age, geography, diet, use of antibiotics, drugs, and sexual behaviors (8, 9). Additionally, sampling methods and the level of bacterial classification applied also impact descriptions of microbial composition (3, 10, 11). While there is much variation in the composition of a healthy microbiome, experts agree that the key to a healthy microbiome lies in the balance of the many different species of microorganisms that exist within the body. Alterations to the gut microbiome may lead to dysbiosis, defined in this review as a significant change in gut microbiome diversity, composition, or function relative to controls. In the GI tract, most residing bacteria are anaerobes, though aerobes and facultative anaerobes are still present (3, 12). The four dominant bacterial phyla in the gut are Firmicutes, Bacteroidota, Actinobacteria, and Proteobacteria, of which *Bacteroides, Clostridium, Faecalibacterium, Eubacterium, Ruminococcus, Peptococcus, Peptostreptococcus*, and *Bifidobacterium* are the most common genera (12). Accordingly, microbial diversity has been used as a key indicator of health and disease.

The role of the microbiome in HIV infection, in particular, is an area of active interest. To date, approximately 38 million people are living with HIV infection worldwide (13). Though antiretroviral therapy (ART) has significantly decreased patient mortality, improving the quality of life for people living with HIV (PLWH) and decreasing HIV-associated comorbidities are areas of top concern. The role of the microbiome in driving some of these comorbidities is becoming increasingly clear. For instance, a number of studies have reported that microbial dysbiosis following HIV infection has been associated with increased inflammation, infection, and decreased gut epithelial barrier integrity (14). Additionally, opioid use, which commonly co-occurs with HIV, is also independently associated with microbial dysbiosis and worsened patient outcomes. Notably, PLWH are likely to have chronic pain and receive higher doses of opioids that put them at increased risk for opioid use disorder (OUD) (15). Of note, though opioids are the gold standard for pain management in the clinical setting, opioid use is associated with poorer outcomes in PLWH (16). Furthermore, high risk behaviors, such as recreational drug use, can put patients at additional risk for both HIV infection and OUD. For these reasons, there has been an increasing focus towards investigating the intersection of PLWH who use opioids, including exploring compositional and functional microbial alterations, with the goal of restoring microbial homeostasis through probiotic intervention, though this area has largely been unexplored. In this review, we aim to explore the intersection between HIV, OUD, and the gut microbiome to investigate how these conditions contribute to dysbiosis and end with an examination of potential therapeutic interventions.

## 2 HIV and alterations to the gut microbiome

### 2.1 Viral alterations to the gut microbiome in PLWH

**Figure 1:**
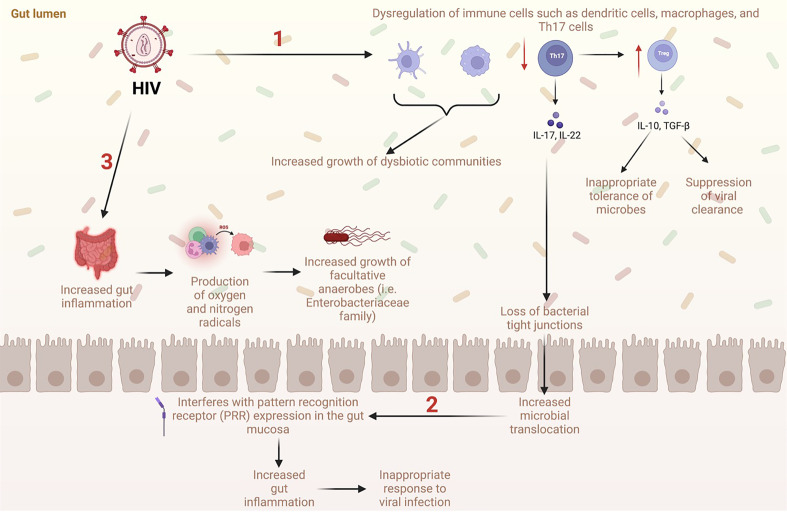
Proposed mechanisms of gut dysbiosis and microbial translocation in the GI tract with HIV (1). HIV causes dysregulation of immune cells such as dendritic cells, macrophages, and Th17 cells, which increases the growth of dysbiotic communities. Additionally, HIV causes a significant decrease in Th17 cells, a subtype of CD4+ T cells, which normally limit microbial translocation. Loss of Th17 cells leads to a decrease in tight junctions in the intestinal epithelium allowing microbes to travel freely between the lumen and systemic circulation. Loss of Th17 cells also causes an increase in Treg cells, which are associated with suppression of viral clearance and inappropriate tolerance of microbes. (2) Increased translocation of microbes into systemic circulation interferes with expression of the pattern recognition receptor (PRR) which ultimately increases gut inflammation, decreases immune response to viral infection, and increases growth of dysbiotic communities. (3) Increased gut inflammation from HIV infection leads to increased production of oxygen and nitrogen radicals. This is associated with increased growth of facultative anaerobes such as *Enterobacteriaceae* family.

In recent years, many studies have detailed the importance of the microbiome in modulating immune function in PLWH. In general, these studies have found an increase in potentially pathogenic bacteria and a decrease in commensal bacteria, which has been associated with heightened inflammation (17–19). Proposed mechanisms of gut dysbiosis with HIV include disruption of microbial pattern recognition sensors, increased reactive oxygen and nitrogen radicals, and dysregulation of immune cells (**Figure 1**) (20). At the phylum level, several studies have reported a shift from *Firmicutes*, which has immune regulatory and probiotic effects, to *Proteobacteria*, which has been hypothesized to have potential pathogenic activity in humans (14, 20–22). In accordance with prior findings of increased systemic inflammation in PLWH, Vujkovic et al. found that bacterial communities that catabolize tryptophan were increased in the plasma of PLWH (20). In these studies, indoleamine 2,3-dioxygenase 1 (IDO-1) was used to assess tryptophan catabolism to downstream metabolites such as kynurenine, kyneuric acid, and anthrallic acid; this process is upregulated in conditions where the immune system is activated (23, 24). Other bacteria such as *Bacteroides*, which is associated with T-regulatory cell function, has consistently been found to decrease, while *Prevotella*, which is associated with pro-inflammatory effects, has been found to increase in both PLWH and rhesus macaques infected with simian immunodeficiency virus (SIV) (3, 25). An increase in potentially pathogenic bacteria, including *Sarcina*, *Staphylococcus, Selenomonas*, and *Tetragenococcus*, has also been found in fecal samples from chimpanzees with SIV (26). Additionally, some have shown that the *Firmicutes/Bacteroides* ratio is increased in PLWH (27, 28). Increases in this ratio have been associated with obesity, irritable bowel syndrome, and *Clostridium difficile* infection (29–31). However, the *Firmicutes/Bacteroides* ratio is also known to increase with age compared to that observed during childhood, with another decline seen in older adults compared to middle-aged adults (32). Mixed results exist regarding changes in the production of immunomodulatory SCFA produced from bacteria in the GI tract of PLWH. Some studies have found no differences in SCFA between PLWH- specifically men who have sex with men (MSM) and negative controls (33, 34). However, others have found that SCFAs are increased in PLWH, a finding largely driven by the SCFA propionate, since other SCFAs such as butyrate and acetate were decreased in this population relative to controls (28).

Different subpopulations of PLWH have been found to have various alterations to their gut microbiome and biomarkers of inflammation. For instance, sexual behavior has been linked to changes in microbial composition in PLWH. It is hypothesized that the shift from *Bacteroides* to *Prevotella* in the MSM population is independent of HIV serostatus. MSM were also found to have increased alpha diversity, which contrasts with findings from other subpopulations living with HIV (35). Additionally, it has been found that levels of inflammatory markers sCD14, sCD163, interleukin 6, and lipopolysaccharide binding protein were already significantly higher in MSM living with HIV before seroconversion compared to controls (33). Interestingly, Chen et al. also found that various microbiota profiles were associated with the progression of HIV to AIDS; in particular, families *Prevotella* and *Victivalla* and species *Bacteroides fragilis* and *Eubacterium cylindroides* were significantly higher in MSM living with HIV who developed AIDS within 5 years than MSM who were AIDS-free for more than 10 years without ART (33). Also, family *Victivallaceae* and species *Prevotella stercorea*, *Coprococcus eutactus*, and *Butyrivibrio crossotus* were significantly higher and *Gemmiger formicilis* and *Blautia obeum* were significantly lower in MSM with HIV who developed AIDS within 5-10 years compared to MSM with HIV who were AIDS-free for more than 10 years without ART (33). Women living with HIV (WLHIV) did not have significantly different microbial diversity measures when compared to women not living with HIV (36). However, it was found that WLHIV had increases in *Ruminococcus* and *Oscillospira* and decreases in *Bifidobacterium* and *Collinsella* when compared to women without HIV. This was further associated with elevated glycerophospholipid levels which could contribute to inflammation and metabolic conditions in WLHIV (37). On the other hand, there was no correlation between *Ruminococcus* and amino acids or biogenic amines, but there was a positive correlation between *Collinsella* which is decreased in WLHIV, and glutamic acid (37) In regard to perinatal HIV infection, mixed results exist regarding microbial diversity. Flygel et al. reported that children living with HIV demonstrated significantly lower alpha diversity and higher beta diversity compared to children without HIV. This change was not associated with differences in CD4 counts or HIV viral load (38). On the other hand, Sessa et al. reported that the alpha diversity indices for perinatally-infected children with HIV were higher than controls (39). ART use for more than 10 years was associated with increased alpha diversity in perinatally-infected individuals living with HIV (38). Additionally, in parallel to adults living with HIV, perinatally infected children living with HIV had higher levels of *Prevotella* present despite ART usage (40).

### 2.2 ART-induced alterations to the gut microbiome in PLWH

Even though ART has been effective in decreasing the viral load, morbidity, and mortality of PLWH (41, 42), ART does not reverse HIV-related gut dysbiosis, leaving patients vulnerable to conditions such as GI and metabolic syndromes associated with gut dysbiosis (42, 43). In fact, up to nearly 75% of PLWH who are on ART experience one of the following GI symptoms with 30% experiencing three or more symptoms: nausea and/or vomiting, dyspepsia, heartburn, diarrhea, constipation, and flatulence (43, 44). Several studies have demonstrated that the gut microbiomes of PLWH using ART differ significantly from healthy controls (9, 17, 22, 45–47). However, the results of these studies are difficult to interpret as it is unclear whether the changes in the microbiome are associated with HIV infection or ART use; additionally, the confounder of sexual behavior is inconsistently accounted for. Compared to studies focused solely on PLWH using ART, studies that included PLWH not using ART found that PLWH using ART have microbiomes more similar to PLWH not using ART than to healthy controls in both the long- and short-term (42, 48). This finding could support that ART does not reverse gut dysbiosis in PLWH (42, 48). While some have reported that dysbiosis persists after ART initiation (49) others have found a decrease in dysbiosis over time (including a decrease in *Prevotella* abundance) (17, 50). On the other hand, those not on ART have been reported to have increased abundance of the bacteria *Pseudomonas aeruginosa* (21, 33), *Enterobacterodales* (51), and *Bacteriodales* (51). Others have also shown that ART causes microbiome changes independent of changes associated with HIV infection (17, 42). For instance, it has been reported that there is decrease in alpha diversity in PLWH who are on ART compared to PLWH not on ART (43); this could imply that ART may independently induce gut dysbiosis since decreased alpha diversity is associated with various diseases such as inflammatory bowel disease (52) and known side effects of ART such as diarrhea are also clinical indices of gut dysbiosis (43). However, mixed results exist regarding the association between ART and alpha diversity. In contrast to the above, McHardy et al. found that the reduction in alpha diversity in PLWH was partially reversed with ART (46). Clarifying this discrepancy could be important as alpha diversity has been shown to correlate with CD4+ T cell count; this is significant since decreases in CD4+ T cell count leave PLWH more susceptible to disease (17). Other confounders in studies addressing ART use and dysbiosis, such as the impact of direct drug metabolism by gut microbes, differences in ART regimen, duration of ART, and the success of ART in reducing the viral load, should also be considered in future studies (42).

## 3 Opioid use results in distinct alterations to the microbiome

### 3.1 Direct opioid induced alterations to the gut microbiome

Approximately 21% to 53% of PLWH are prescribed opioids and often at higher doses (15). PLWH have an increased incidence of chronic pain with up to 90% of PLWH experiencing chronic pain (53). Management of chronic pain in PLWH is difficult due to interactions with ART, increased side effect profiles, and considerations of comorbid conditions such as OUD (53). Studies have shown that there is a higher risk of OUD in PLWH, although exact estimates are unknown (15). Given that opioid use has been strongly tied to microbial dysbiosis, and microbial dysbiosis has been linked to a variety of health impairments, opioid use in PLWH poses an additional set of challenges.

While limited data exist regarding microbiome changes in humans with OUD due to confounding variables like polydrug use among people with OUD (54–56), two studies found increased species diversity in those using opioids (57). Specifically, Xu et al. (57) found an abundance of *Thauera, Paracoccus*, and *Prevotella* in fecal samples from humans with substance use disorder (SUD) compared to healthy controls. In humans, opioid use has been shown to increase microbial diversity partially attributed to opioid-induced increased GI transit time. This GI stasis could allow for more microbial growth in the colon (58). Additionally, Barengolts et al. showed an increase in *Bifidobacteria* in opioid users compared to non-users (59). Microbial dysbiosis following opioid use has also been associated with alterations in cellular and metabolic pathways in humans. Species involved with translation, DNA replication and repair, and cell growth and death were found to be increased in those with SUD (57). The clinical implications of these findings are unclear, but have shown that dysbiosis is associated with the cardiovascular and digestive systems, which are common comorbidities of OUD (57). Lastly, several studies have shown increased intestinal barrier permeability with opioid use. opioid antagonists have been demonstrated to promote mucosal healing in people with Crohn’s disease (60), bacterial translocation (61), and increased risk of infection related to the GI system (62, 63).

**Figure 2:**
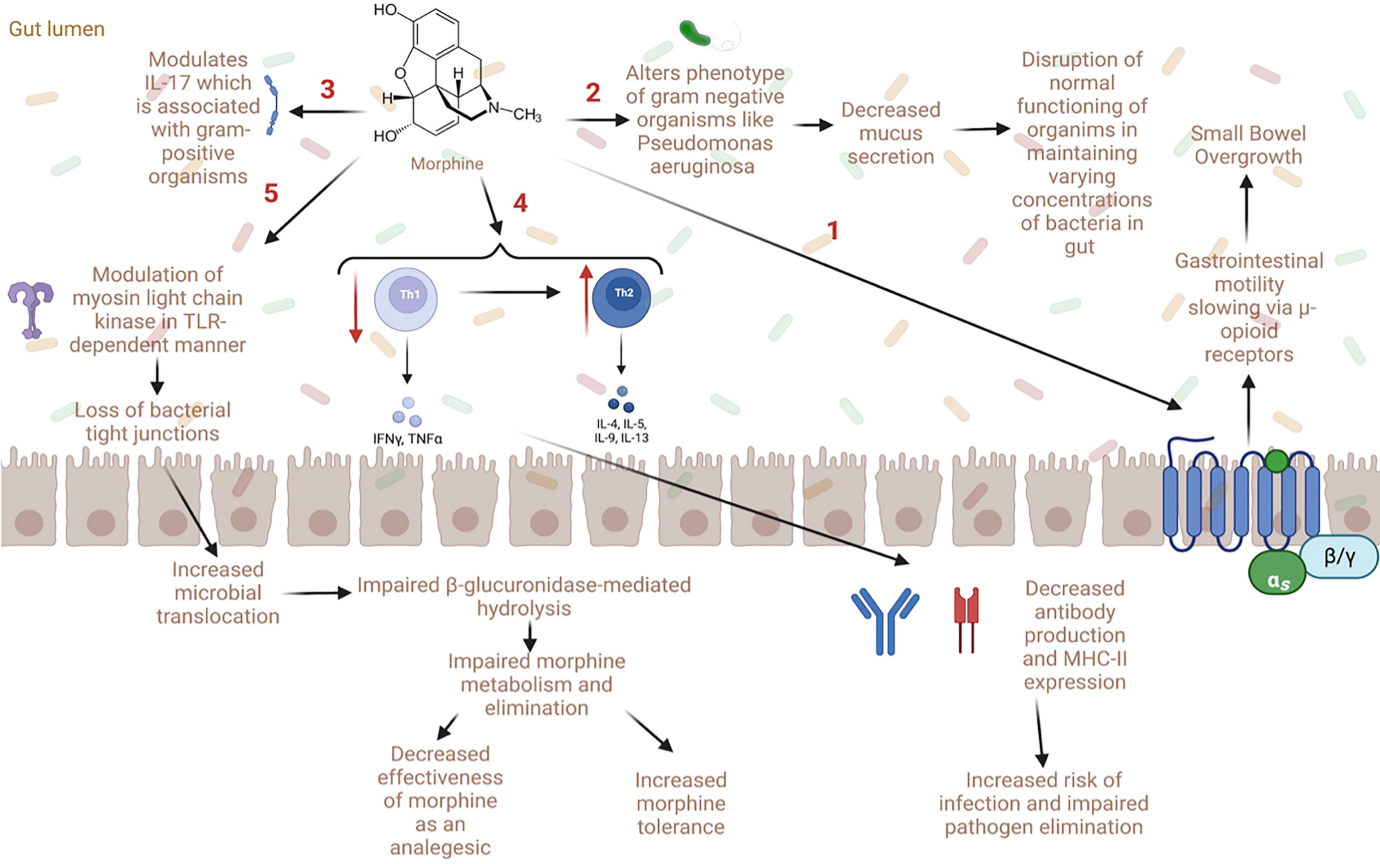
Proposed mechanisms of gut dysbiosis and microbial translocation with opioid use (1) Opioid use is associated with decreased gastrointestinal motility *via* direct action on μ-opioid receptors. This can lead to small bowel overgrowth, gut dysbiosis, and microbial translocation. (2) Some opioids (e.g., morphine) have been shown to directly alter the phenotype of gram-negative organisms such as *Pseudomonas aeruginosa* which causes decreased mucus secretion in the gut, altering microbial homeostasis. (3) Opioids can also worsen sepsis *via* modulation of IL-17 by gram-positive organisms. (4) Opioids also cause a decrease in Th1 and an increase in Th2 cells which lead to decreased antibody production and MHC-II expression; this increases the risk of infection and decreases pathogen elimination. (5) Lastly, opioids modulate myosin light chain kinase in a toll-like receptor dependent manner which causes loss of bacterial tight junctions and impaired gut barrier function. This increases microbial translocation and impairs β-glucuronidase-mediated hydrolysis, which can in turn impair morphine metabolism and elimination. Notably, alterations in opioid metabolism can decrease the effectiveness of morphine as an analgesic agent and increase morphine tolerance.

In general, preclinical models of opioid use have cited an increase in potentially pathogenic microbiome species (64, 65); in particular, an increase in gram-positive bacteria, including *Enterococcaceae, Staphylococcaceae, Bacillaceae, Streptococcaceae*, and *Erysipelotrichaceae*, all belonging to the phylum Firmicutes, has been reported (56, 65). Mice with chronic opioid exposure also were found to have a decreased abundance of the beneficial bacterial order *Bacteriodales, Clostridiales, and Lactobacillales* (66). From these studies, some have proposed that gut dysbiosis in those with OUD is driven by slowed GI motility *via* μ-opioid receptors, modulation of IL-17 by gram-positive organisms, and/or altered phenotypes of facultative anaerobes using evidence from both human and mice studies (**Figure 2**) (56, 63, 67). Conflicting results regarding the impact of opioids on species diversity has been reported in animal studies. For instance, two studies found increases in diversity and community richness (65, 68), and one study found a decrease in species diversity (69). Additionally, increases in bacterial taxa involved with amino acid metabolism have been reported during opioid administration, extinction, and reinstatement (68). These taxonomical changes were associated with alterations in protein synthesis, gene transcription, cell signaling, and synaptic plasticity (68). Additionally, decreases in bacteria and bacterial products involved in the bile acid transformation pathway, such as *Bacteroidetes* and *Bilophila*, have been reported with opioid use (65, 68–70). Specifically, significant changes in bile acid and the morphine-3-glucuronide/morphine biotransformation pathways in the gut were found in a morphine-murine model (69). Changes in species associated with bile acid metabolism have also been reported in humans using opioids (70). Importantly, bile acid metabolism is associated with the metabolism, elimination, and enterohepatic recirculation of opioids which contributes to the bioavailability of morphine and its effectiveness as an analgesic (69). Thus, a decrease in species associated with bile acid metabolism could be connected to opioid tolerance over time and contribute to opioid misuse and OUD (69). Gicquelais et al. also found a decreased abundance of *Roseburia* which is a SCFA producer (70). This reduction in SCFAs could be due to decreases in butyrate-producing bacteria (BPB) (71). SCFAs act on various free fatty acid receptors to regulate the production of eicosanoids, chemokines, and cytokines involved in inflammatory responses (72). Collectively, these studies show profound changes to the gut microbiome and metabolome with opioid use.

### 3.2 Effect of medication assisted treatments for OUD on microbial dysbiosis

Great strides have been made in the treatment of OUD and it is generally agreed upon that MAT leads to positive outcomes for patients (73). However, research on the effect of MAT on the gut microbiome is limited due to the novelty of OUD research and the difficulty of studying those with OUD. Currently, only two studies to date detail interactions between medication assisted treatments (MATs) such as methadone and buprenorphine and the gut microbiome.

First, Gicquelais et al. (70) studied gut microbiota characteristics related to the use of opioid agonists and antagonists among people receiving outpatient addiction treatment. In their studies, they reported a decrease in microbial species diversity in patients who used opioid agonists (without antagonists), a decreased abundance of *Bacteroidetes* enterotypes, and decreased *Roseburia* and *Bilophila*, which are butyrate and bile acid-related species, respectively. However, there were no differences in the gut microbiome in those using opioid agonist-antagonist combinations (e.g., buprenorphine-naloxone), antagonists alone (e.g., naltrexone, or those using neither opioid agonists nor antagonists. This study had several limitations including a smaller sample size and lack of healthy controls. Additionally, as nearly all measures were self-reported, this study was subject to selection bias. Still, this was the first study to describe enterotypes among humans with OUDs and allows for comparisons to other disorders to identify common mechanisms underlying dysbiosis.

Next, Cruz-Lebron et al. also studied the impact of methadone maintenance treatment on the gut microbiome, metabolome, and intestinal barrier integrity. This group found that methadone-treated individuals had decreased fecal bacterial diversity as measured by beta diversity as well as decreased evenness and richness (74). Additionally, it was found that bacteria involved with the production of SCFAs, mucus degradation, and maintenance of intestinal barrier integrity were decreased in those who used methadone, which could leave those individuals at risk for conditions associated with decreased intestinal barrier integrity (75).

Ultimately, the limited number of studies do not permit generalizations on the effect of MAT on microbial dysbiosis, and further work should be done to investigate the impact of MAT on the microbiome. However, the currently available data suggests that MAT also induce alterations to the gut microbiome; whether these alterations are less severe than those imposed by other opioid agonists themselves still needs to be explored in larger well-designed studies using appropriate control groups.

## 4 HIV, opioids, and alterations to the gut microbiome

Understanding how HIV and opioid use, in combination, impact the microbiome is of growing importance, with the opioid epidemic leaving no populations untouched and opioids still widely used in clinical settings for pain management (76). In addition, those living with both HIV and OUD are more likely to receive suboptimal HIV testing and delayed entry into HIV care and treatment, and less likely to be adherent to ART treatment (16). Of note, opioids and MAT can also interact with and reduce the efficacy of ART (16). Thus, detailing interactions between HIV and opioid use is paramount to improving patient care and coordinating disease management.

Many studies have separately examined differences in microbial composition in animal and human models of HIV and opioid use and have concluded that both of these conditions significantly impact the microbiome. However, with accumulating evidence of the interaction of opioids and HIV infection, it is logical to assess the compounding effect they may enact together on the gut ecosystem. Of the few studies that exist, there is strong evidence that HIV in conjunction with opioid use can cause microbial dysbiosis that is distinct from the dysbiosis seen in HIV/SIV infection or those with opioid use alone (77–79). Additionally, this dysbiosis, like that seen with HIV infection or opioid use alone, is associated with inflammation (77–79). To date, only three preclinical studies have described microbial changes with HIV and opioid use; one study assessed dysbiosis in humanized mice injected with HIV and then administered morphine, and two studies assessed dysbiosis in rhesus macaques treated with morphine followed by SIV infection (**Table 1**) (77–79).

**Table 1:** Summary of preclinical studies detailing HIV and opioid-induced microbial alterations.

Author Name, Year	Methods	Main Findings	Supplementary Findings
**Meng et al.** (79)	n = 48, with 12 controls, 12 morphine treated animals, 12 humanized BLT mice injected with HIV, and 12 humanized BLT mice injected with HIV and subsequently administered morphine, aged 17-19 weeks Treatment: I.V. injection of HIV particles (10 ng of calculated p24 antigen/mice) for 4 weeks; HIV + morphine group: 75 mg slow-release morphine pellet implantation 21 days after HIV infection Region analyzed: Intestinal contents from cecal-colonic region	HIV/morphine group: ↑ *Firmicutes, Proteobacteria* ↓ *Bacteroides, Actinobacteria*, *Tenericutes*, alpha diversity ↓ *Lachnospiraceae, Ruminococcaceae*, and *Muribaculaceae*	↓ gut epithelial barrier integrity in HIV/morphine group
**Johnson et al.** (77)	N = 10 out-bred, Indian-origin rhesus macaques randomly subdivided into saline (n = 5) or morphine (n = 5) treatment groups. Treatment: 7 weeks pre-treatment with 6 mg/kg saline/morphine, followed by I.V. inoculation with 200 TCID_50_ of SIVmac251 Region analyzed: fecal samples at necropsy	HIV/morphine group: ↓ *Firmicutes, Ruminococcacea*, butyrate-producing bacteria, alpha diversity. ↑ *Bacteroidetes* such as *Prevotellaceae* Positive correlation between *Lachnospiraceae* and butyrate-producing bacteria	Morphine group: ↓ butyrate-producing bacteria, which is not associated with ↓ in *Ruminococcacea*
**Sindberg et al.** (78)	N = 14 out-bred, male, Indian-origin rhesus macaques aged 3-4 years of age divided into morphine alone (n=4), SIV alone (n=4), SIV + morphine (n=4), or saline (n=2) groups Treatment: intramuscular injections t.i.d with 2 mg/kg (week 1), 3 mg/kg (week 2), and 4 mg/kg (week 3) of saline/morphine 70 days before SIV inoculation (10^4^ plaque forming units of virus SIVmacR71/17E) Region analyzed: longitudinal analysis of fecal samples	Microbial diversity unaltered by SIV and morphine. No significant difference in microbial composition in SIV infected group alone. Morphine and Morphine/SIV groups: ↓ *Methanobacteriaceae*, *Streptococcaceae*, and *Ruminococcaceae* family microbes at very early time points of infection (3 weeks after infection)	Select primary bile acids ↓ in morphine animals. Secondary bile acids ↑ in morphine + SIV animals.

Meng et al. reported gut microbial dysbiosis in a mouse model of HIV infection subsequently administered morphine (79). It was found that the alpha diversity in mice treated with HIV and morphine (HIV/morphine) was significantly lower than in placebo mice treated with morphine or HIV alone. This could indicate an additive effect of HIV and morphine on microbiome dysbiosis. In the HIV/morphine group, there was a greater abundance of *Firmicutes* and *Proteobacteria* and a lower abundance of *Bacteroides, Actinobacteria*, and *Tenericutes*. Interestingly, a depletion of *Lachnospiraceae, Ruminococcaceae*, and *Muribaculaceae* was found in the same group (79). These bacteria are commonly associated with SCFA production in the gut, which is essential in modulating inflammation *via* histone deacetylase inhibition (80). In fact, SCFAs, like butyrate, are the preferred energy substrates for colonocytes, and decreased butyrate production is associated with inflammatory conditions like Crohn’s disease and ulcerative colitis (81, 82). Additionally, butyrate is responsible for upregulating the tight junction protein Claudin-1, which promotes intestinal epithelial barrier integrity (83). This study also found that HIV/morphine mice had significant epithelial intestinal damage. Additionally, *Staphylococcus* was found to be increased in mice with HIV infection, and *Enterococcus* was found to be increased in HIV/morphine mice. These are common pathogens associated with bacteremia in PLWH (84) and in those using opioids (85). *Lactobacillus*, a common microbe of the intestine that is anti-apoptotic and anti-inflammatory, was also found to be depleted (86). How these findings translate to humans remains to be elucidated.

In accordance with Meng et al., Johnson et al. reported that rhesus macaques that were first administered morphine, injected with SIV, and then given ART (SIV/ART/morphine) to suppress viral load had decreased alpha diversity in fecal samples collected. Notably, there was decreased *Firmicutes*, in particular *Ruminococcacea* (77). It was hypothesized that the *Ruminococcacea* depletion was the result of morphine treatment, since there was a trend toward decreased BPB in the SIV/ART/morphine group compared to SIV/ART alone. Interestingly, the authors found that the decreased BPB was likely not due to the *Ruminococcacea* since there was no correlation between *Ruminococcacea* and BPB. However, there was a positive correlation between *Lachnospiraceae* and BPB which could indicate that, of the SCFA-associated bacteria found to be decreased in the SIV/ART/morphine macaque, *Lachnospiraceae* could be important in SCFA production. Johnson et al. also found that SIV/ART/morphine primates had increased *Bacteroidetes* such as *Prevotellaceae*; this contrasts with Meng et al. who reported decreased *Bacteroidetes*. Still, increases in *Prevotella* are often associated with increased risk of other inflammatory conditions such as rheumatoid arthritis and colitis due to changes in mucosal immune responses (87, 88). While this study did not assess temporal changes in the gut microbiome, future studies would benefit from this evaluation.

In contrast to the above studies, rhesus macaques that were first administered morphine and then injected with SIV did not have significantly different alpha diversity in the microbiome during early SIV infection when compared to the placebo group, the SIV group, or the morphine group (78). Additionally, there was no significant difference in microbial composition in the group with SIV infection alone. The group with morphine administration had increased *Methanobacteriaceae* of the domain Archaea and decreased *Streptococcaceae* and *Ruminococcaceae* family microbes. A similar finding was seen in SIV/morphine primates, which could indicate that morphine primarily contributes to dysbiosis in early infection. In accordance with both Meng et al. and Johnson et al., Sindberg et al. also found a decrease in *Ruminococcaceae*. These differences in microbial composition in the morphine group and the SIV/morphine group were associated with altered metabolites such as bile acids, sphingolipids, and serotonin. Bile acid metabolism and dysregulation of this pathway associated with microbiome dysbiosis is also seen in many studies assessing dysbiosis in opioid use alone, which could indicate the important role of opioid metabolism in dysbiosis during early HIV infection (65, 68–70). Bile acids play a role in bacterial translocation, which is associated with HIV replication and might occur earlier in PLWH who also use opioids (69). Thus, the bile acid metabolism pathway could serve as a potential target for decreasing HIV replication.

**Figure 3:**
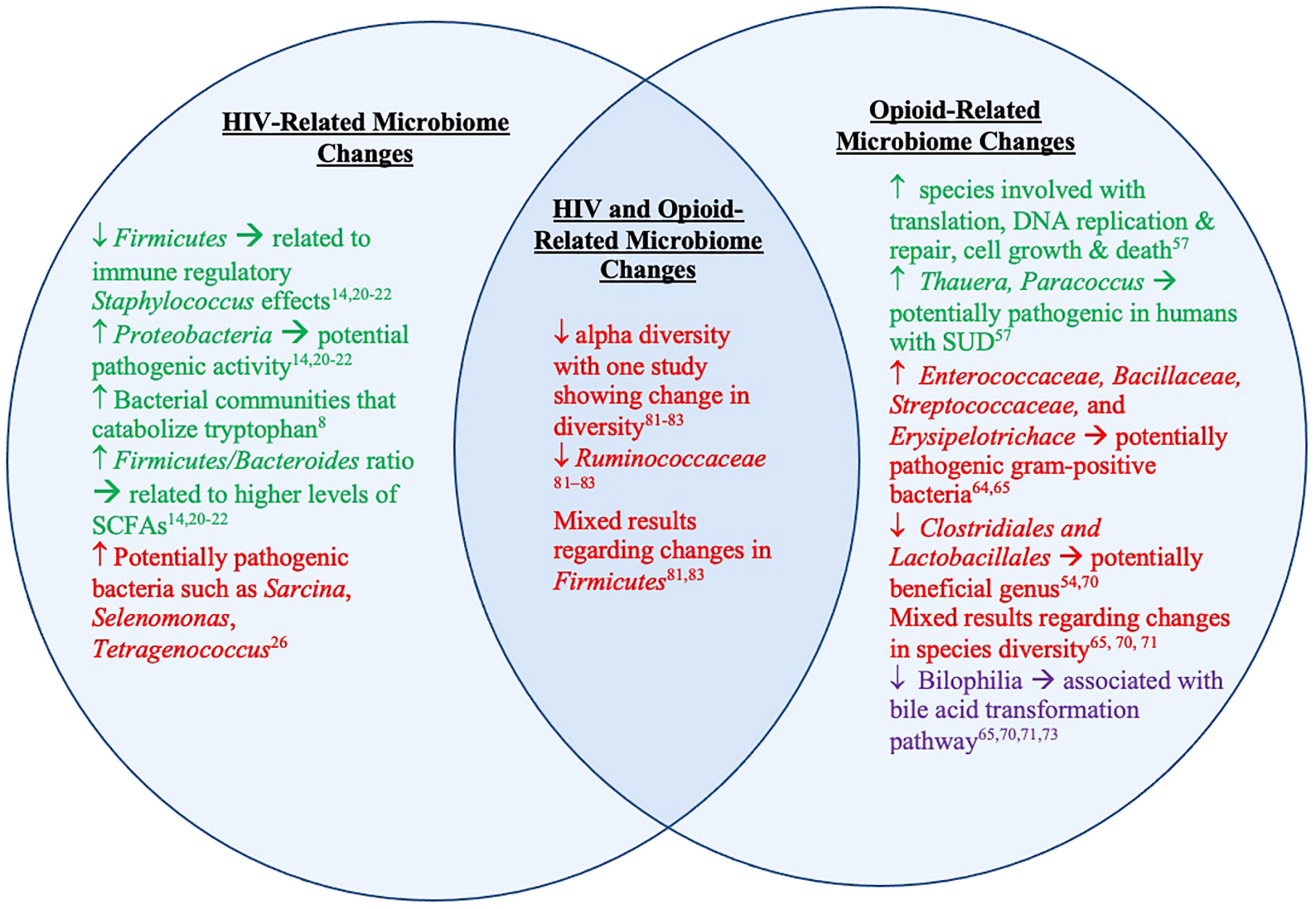
Microbiome and metabolite changes in clinical and preclinical studies of HIV and opioid use. The left represents microbiome and metabolite changes in PLWH and animal models of HIV/SIV. The right represents microbiome and metabolite changes in those with OUD and animal models of opioid use/OUD. The middle represents the overlapping microbiome detailed in existing animal models of both HIV/SIV and opioid use. The text in green represents information from studies done in humans. The red text represents information from studies done in animal models of HIV/SIV and opioid use. The text in purple represents information from both studies done in humans and animals.

Together these studies suggest that HIV and opioid use together can create a state of microbial dysbiosis that is distinct from dysbiosis caused by either state alone (**Figure 3**). It is imperative to highlight that there are no human studies in this field precluding generalizations to humans. Nonetheless, animal models are known to recapitulate human conditions. For example, humanized mouse models of HIV model HIV-1 infection of human cells *in vivo*. Similarly, research in SIV infection in macaques has evolved to developing SIV/HIV hybrid viruses to further resemble of the viruses to one another, which is currently the most widely accepted model of HIV (89). Likewise, SUD animal models have shown great validity due to similarities between neurochemical and neuroanatomical substrates involved in drug-intake behavior (90). Specifically, mice models have been shown to be translationally relevant and useful for studying opioid use disorder. Slivicki et al. showed that mice voluntarily self-administer oxycodone; some mice even demonstrated drug taking that is resistant to aversive consequences (91), similar to OUD. While animal models have helped elucidate mechanisms, more studies that are focused on human subjects are needed.

## 5 Treatment of dysbiosis in PLWH with OUD

**Figure 4:**
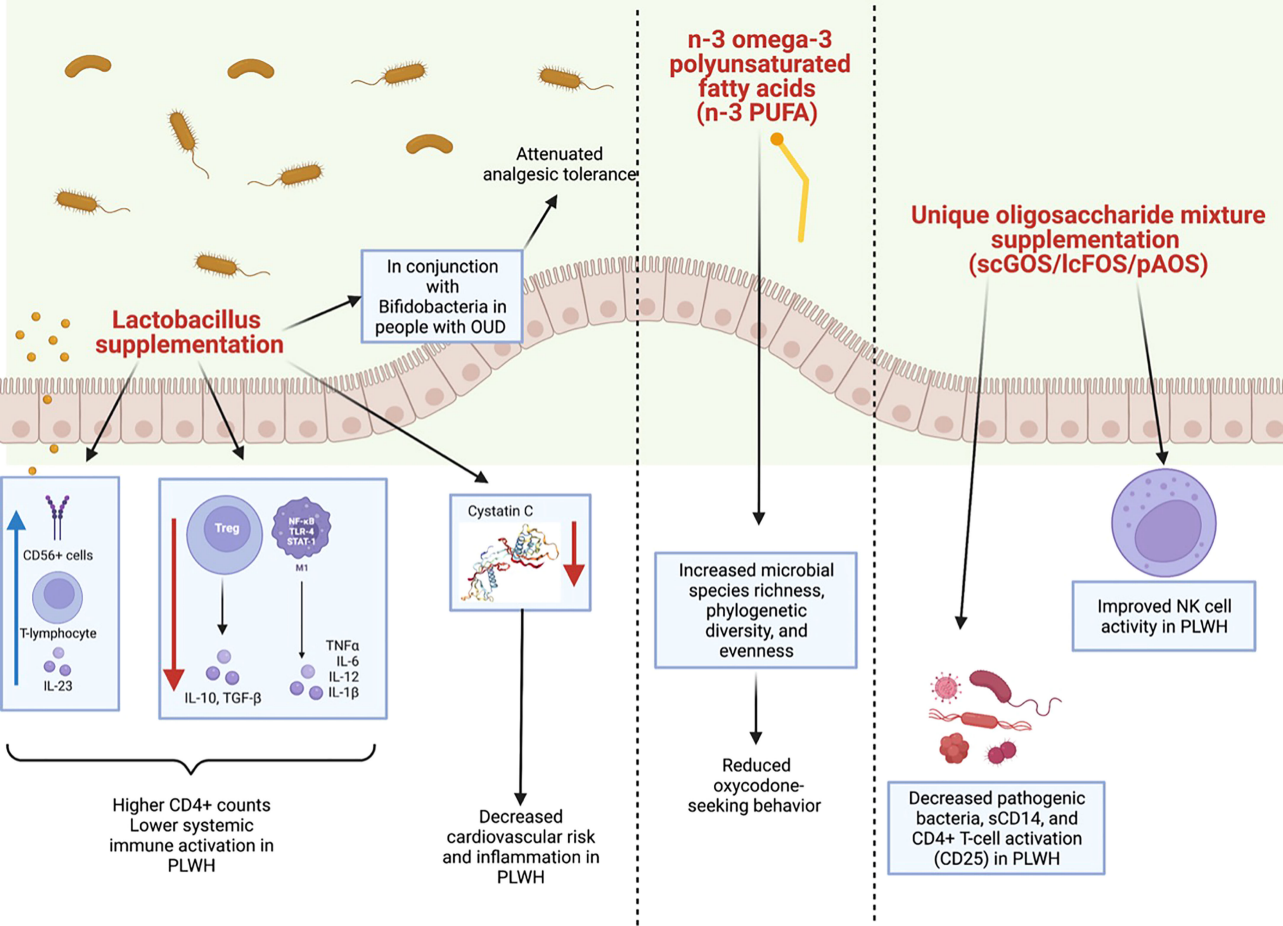
Summary of clinical and preclinical studies detailing the impact of probiotics with HIV or opioid use. (Left panel) *Lactobacillus* supplementation has been shown to have a positive impact on the microbiomes of both clinical and preclinical models of HIV or opioid use. In PLWH, *Lactobacillus* supplementation has been shown to increase CD56+ cells, T-lymphocytes, and IL-23 and to decrease levels of TGFβ, IL-10, IL-12 and IL-1β. This is thought to lower systemic immune activation and to increase CD4+ counts in PLWH. *Lactobacillus* supplementation also decreases cystatin C (image from https://www.sinobiological.com/resource/cystatin-c/proteins) which in turn, decreases cardiovascular risk and inflammation in PLWH. *Lactobacillus* supplementation in conjunction with *Bifidobacteria* supplementation also has been shown to attenuate analgesic tolerance in morphine-treated mice. (Middle panel) Additionally, in preclinical models of chronic opioid use, an n-3 omega-3 polyunsaturated fatty acids (n-3 PUFA) enriched diet increased species richness, phylogenetic diversity, and evenness, as well as decreased oxycodone-seeking behaviors. (Right panel) Lastly, a unique oligosaccharide mixture supplementation including short-chain galacto-oligosaccharides (scGOS), long-chain fructo-oligosaccharides (lcFOS), and pectin hydrolysate derived acidic-oligosaccharides (pAOS) in PLWH has been shown to decrease pathogenic bacteria, sCD14, and CD4+ T-cell activation in PLWH, as well as to improve NK cell activity.

Alterations to the microbiome observed in those with HIV, OUD, or both could implicate significantly adverse downstream effects. While there are no papers to date about treating dysbiosis in PLWH with co-occurring OUD, there have been studies that have investigated microbial restoration in people with either HIV or OUD. Specifically, therapies such as prebiotic supplementation and fecal microbiota transplantation (FMT) have been attempted and investigated (**Figure 4**).

### 5.1 Treatment of dysbiosis in PLWH

Prebiotic, probiotic, and symbiotic supplementation has had some success in modulating gut dysbiosis in PLWH. Interestingly, enriching the microbiome with *Lactobacillus* has been shown to be associated with higher CD4 counts and lower immune activation in peripheral blood of PLWH (47, 92). Specifically, *Lactobacillus* supplementation has been linked to increased T-lymphocytes, CD56+ cells, and serum IL-23 in peripheral blood of PLWH (93). Supplementation has also been associated with decreased mRNA levels of TGFβ, IL-10, IL-12 and IL-1β (93). This study also found a decrease in cystatin C, which is associated with lower inflammation and cardiovascular risk (93). These studies are in line with the finding that ART-treated individuals who fail to have an immune response (defined by a CD4 count < 200) have lower levels of anti-inflammatory microbes such as *Lactobacilli* and increased inflammatory markers such as IL-6 sCD14, and LPS (94–96). While ART did not improve gut microbiome dysbiosis, probiotic supplementation was shown to significantly decrease markers of inflammation and of microbial translocation (97). However, conflicting studies exist regarding probiotics in PLWH who are not on ART. Gori et al. found that probiotic supplementation in combination with a unique oligosaccharide mixture including short-chain galacto-oligosaccharides (scGOS), long-chain fructo-oligosaccharides (lcFOS), and pectin hydrolysate derived acidic-oligosaccharides (pAOS) significantly decreased pathogenic bacteria, sCD14, and CD4+ T-cell activation (CD25) and improved NK cell activity in this population (98). However, limited or no success with probiotic supplementation has also been evenly reported. For instance, Kim et al. found no significant differences in immune activation markers of 40 PLWH not on ART after probiotic supplementation with a commercially available supplement that included 900 bacterial colony forming units (99).

Fecal microbiota transplantation (FMT) to treat gut dysbiosis in PLWH has also had mixed results. To date, only three studies have investigated the use of FMT to treat dysbiosis in PLWH (100–103). Vujkovic-Cvijin et al. selected control donors for FMT who had a low abundance of *Proteobacteria* and a high abundance of *Bacteroides* in their fecal sample. It was found that PLWH had a microbiome composition that resembled their donors at two and four weeks; however, this shift was lost by week eight. At that point, the microbiome of PLWH was more like their microbiome before FMT. Additionally, no changes in HIV-associated inflammation markers such as plasma levels of immune activation markers including IL-6 or sCD14 were found over time (101). Of note, this study had a small sample size with only six participants and two controls. Similarly, Serrano-Villar et al. selected donors for FMT who had a low abundance of *Prevotella* and a high abundance of *Bacteroides* (102). The donors were also all on effective ART therapy. It was found that there was a significant decrease in intestinal damage assessed by lipopolysaccharide-binding protein and intestinal fatty acid binding protein at week four in PLWH who received FMT. However, similar to Vujkovic-Cvijin et al.’s study, there were no changes in inflammatory markers or disease progression markers associated with HIV such as CD4+ T-cells, CD8+ T-cells, CD4/CD8 ratio, sCD14, IP-10, sCD163, sTNFR-II, D-Dimer, and lipoteichoic acid (102).

Finally, Hensley-McBain et al. studied FMT in non-humans with SIV infection. Stool samples from SIV-negative animals were transplanted into SIV-positive animals. It was found that the animals who received antibiotics before transplantation had decreased CD4+ T-cells, increased Th17 and Th22, and increased engraftment and uptake of FMT. There was also an incremental increase in alpha diversity that was seen until week 6. However, after this time point, this increase diminished and diversity returned to baseline levels (103). While FMT interventions in PLWH has been limited in its success, there has been more success with FMT use in PLWH who have *Clostridiales difficile* infection (104). Additionally, some studies suggest that the use of antibiotics could improve the uptake of FMT (103, 105, 106). However, much more work is needed in this area, with specific attention paid to maintaining successful engraftment for long-term improvements in PLWH.

### 5.2 Treatment of dysbiosis with OUD or models of opioid-dependency

With regards to OUD-associated dysbiosis, prebiotics and dietary supplementation seem to be promising treatments. Zhang et al. found that prebiotics enriched with *Bifidobacteria* and *Lactobacillaeae* attenuated analgesic tolerance in morphine-treated mice (94). Increasing certain metabolites has also proved to be an important modulator of the gut microbiome. In one study, an omega-3 polyunsaturated fatty acids (n-3 PUFA) enriched diet was found to reduce oxycodone-seeking behaviors in a mouse model of chronic opioid administration (107). Specifically, Hakimian et al. found that n-3 PUFA supplementation increased species richness, phylogenetic diversity, and evenness (107) N-3 PUFA is found in fish and plants and has been shown to have anti-inflammatory and anti-anxiolytic properties (108, 109), which is relevant as anxiety is often a co-morbid condition in those with SUD (110). Modulation of the microbiome using antibiotics has also been shown to improve dysbiosis in rodent models of opioid abuse. Lee et al. found that intermittent morphine treatment in mice was associated with microglial activation, hyperalgesia, and impaired reward response. When antibiotics (ABX) were used to deplete gut microbiota, reduced opioid analgesic potency was observed. Colonization of these ABX-treated mice with a control microbiota resulted in restoration of microglial activation (54). Kang et al. further found that ABX could actually prevent chronic morphine-induced increases in gut permeability, colonic mucosal destruction, colonic IL-1β expression, and the development of antinociceptive tolerance to chronic morphine (66). Collectively, these studies show the profound role of the gut microbiota in modulating analgesic potency, drug tolerance/reward, gut barrier integrity, and immune activation. However, widespread use of antibiotics to modulate gut-microbiota-mediated effects is noted to be impractical in the clinical setting, with microbial resistance becoming a growing public health concern.

Lastly, few studies have investigated FMT in the treatment of OUD-associated dysbiosis. In a preclinical study, Thomaz et al. examined how FMT influences opioid withdrawal, using number of jumps as a somatic sign of withdrawal in mice, and evaluated whether FMT from morphine-treated or saline donors could lessen withdrawal symptoms (111). Their results demonstrated that morphine-dependent mice which received FMT from morphine-treated donors exhibited fewer naloxone-precipitated jumps than those which had saline-treated donors (111). Additionally, broad-spectrum ABX reduced somatic signs of withdrawal in mice, whereas probiotics and a gut permeability inhibitor called ML-7 did not cause a significant change in physical withdrawal symptoms (111). These promising results show that therapeutic manipulations of the gut microbiome may reduce opioid-associated comorbidities.

## 6 Conclusion

In this review, we have described how HIV and OUD can impact the gut microbiome and how alterations to the gut microbiome can influence HIV disease progression. Investigating how HIV in conjunction with OUD impacts the gut microbiome is greatly warranted because of the high co-occurrence of HIV and OUD, growing rates of chronic pain in PLWH, and increased rates of prescription opioid use in PLWH. However, few studies exist regarding this topic. All three studies that exist report various microbial changes in animal models of HIV that were administered opioids. These bacteria are involved with modulating immune function, decreasing colonic inflammation, and maintaining gut epithelial barrier integrity in healthy individuals.

It is imperative to acknowledge the various limitations of this review. While the effect of either HIV or opioid use alone on the microbiome has been studied extensively, very few articles have looked at their combined effect. Additionally, the few that have done so have been carried out in animal models alone. Evaluation of microbiome compositional and functional shifts in PLWH with OUD are necessary. Secondly, there is great inconsistency in the way microbial changes are described in literature, making comparisons of existing studies difficult. Additionally, while animal studies can carefully control for confounding variables, including polysubstance use that typically occurs with PLWH with OUD, differences in species used amongst animal studies further complicate gut microbiome comparisons and limit generalizations to humans.

Still, our review is the first to look at the interaction of HIV and opioids and serves to elucidate a knowledge gap for future human studies. A review of existing studies has shown that changes in the gut microbiome from HIV in conjunction with opioid use are distinct from changes observed in HIV or opioid use independently. These changes are also associated with increased inflammation and decreased gut epithelial barrier integrity. While mixed results exist regarding treating dysbiosis with microbial restoration in PLWH or in those with OUD, larger well-defined studies that can extend microbial engraftment or delivery of metabolites hold much promise and should still be explored. Future studies should further investigate how manipulating the microbiome can impact key characteristics of HIV and OUD which have not been previously explored, such as CD4 counts, viral load, opioid seeking behavior, and neuroinflammation, for example. Additionally, investigations into how ART and MAT impact the microbiome, cellular processes and metabolism are warranted.

## Author contributions

SS: Conceptualization, Methodology, Creating figures, Writing-original draft, Writing-review & editing. YA: Conceptualization, Methodology, Writing-review & editing. DG: Writing-review & editing, Assisting with figures. RKD: Writing-review & editing, Visualization. SR: Writing-review & editing, Supervision, Project administration, Funding acquisition. All authors contributed to the article and approved the submitted version.

## References

[1] Clemente JC Ursell LK Parfrey Wegener L Knight R. The impact of the gut microbiota on human health: an integrative view. Cell (2016) 148:1258–70. doi: 10.1016/j.cell.2012.01.035.The PMC5050011 22424233

[2] Amon P Sanderson I. What is the microbiome? Arch Dis Child Educ Pract Ed (2017) 102:258–61. doi: 10.1136/archdischild-2016-311643 28246123

[3] Bandera A De Benedetto I Bozzi G Gori A. Altered gut microbiome composition in HIV infection: Causes, effects and potential intervention. Curr Opin HIV AIDS (2018) 13:73–80. doi: 10.1097/COH.0000000000000429 29045252

[4] Ventura M Turroni F Motherway MOC MacSharry J van Sinderen D. Host-microbe interactions that facilitate gut colonization by commensal bifidobacteria. Trends Microbiol (2012) 20:467–76. doi: 10.1016/j.tim.2012.07.002 22902802

[5] Krantis A. GABA in the mammalian enteric nervous system. Physiology (2000) 15:284–90. doi: 10.1152/physiologyonline.2000.15.6.284 11390928

[6] Aragón IM Herrera-Imbroda B Queipo-Ortuño MI Castillo E Del Moral JSG Gómez-Millán J. The urinary tract microbiome in health and disease. Eur Urol Focus (2018) 4:128–38. doi: 10.1016/j.euf.2016.11.001 28753805

[7] Reynoso-García J Miranda-Santiago AE Meléndez-Vázquez NM Acosta-Pagán K Sánchez-Rosado M Díaz-Rivera J. A complete guide to human microbiomes: Body niches, transmission, development, dysbiosis, and restoration. Front Syst Biol (2022) 2:951403. doi: 10.3389/fsysb.2022.951403 PMC11238057 38993286

[8] Tuddenham S Koay WL Sears C. HIV, Sexual orientation, and gut microbiome interactions. Dig Dis Sci (2020) 65:800–17. doi: 10.1007/s10620-020-06110-y PMC7301749 32030625

[9] Noguera-Julian M Rocafort M Guillén Y Rivera J Casadellà M Nowak P. Gut microbiota linked to sexual preference and HIV infection. EBioMedicine (2016) 5:135–46. doi: 10.1016/j.ebiom.2016.01.032 PMC4816837 27077120

[10] Tang Q Jin G Wang G Liu T Liu X Wang B. Current sampling methods for gut microbiota: A call for more precise devices. Front Cell Infect Microbiol (2020) 10:151. doi: 10.3389/fcimb.2020.00151 32328469 PMC7161087

[11] Ingala MR Simmons NB Wultsch C Krampis K Speer KA Perkins SL. Comparing microbiome sampling methods in a wild mammal: Fecal and intestinal samples record different signals of host ecology, evolution. Front Microbiol (2018) 9:803. doi: 10.3389/fmicb.2018.00803 29765359 PMC5938605

[12] D’Angelo C Reale M Costantini E. Microbiota and probiotics in health and HIV infection. Nutrients (2017) 9(6):615. doi: 10.3390/nu9060615 28621726 PMC5490594

[13] Govender RD Hashim MJ Khan MA Mustafa H Khan G. Global epidemiology of HIV/AIDS: A resurgence in north America and Europe. J Epidemiol Glob Health (2021) 11:296–301. doi: 10.2991/jegh.k.210621.001 34270183 PMC8435868

[14] Dillon SM Lee EJ Kotter CV Austin GL Dong Z Hecht DK. An altered intestinal mucosal microbiome in HIV-1 infection is associated with mucosal and systemic immune activation and endotoxemia. Mucosal Immunol (2014) 7:983–94. doi: 10.1038/mi.2013.116 PMC4062575 24399150

[15] Cunningham CO. Opioids and HIV infection: From pain management to addiction treatment. Top Antivir Med (2018) 25(4):143–6. PMC5935219 29689538

[16] Cernasev A Veve MP Cory TJ Summers NA Miller M Kodidela S. Opioid use disorders in people living with HIV/AIDS: A review of implications for patient outcomes, drug interactions, and neurocognitive disorders. Pharmacy (2020) 8:168. doi: 10.3390/pharmacy8030168 32932786 PMC7559328

[17] Nowak P Troseid M Avershina E Barqasho B Neogi U Holm K. Gut microbiota diversity predicts immune status in HIV-1 infection. Aids (2015) 29:2409–18. doi: 10.1097/QAD.0000000000000869 26355675

[18] Deusch S Serrano-Villar S Rojo D Martínez-Martínez M Bargiela R Vázquez-Castellanos JF. Effects of HIV, antiretroviral therapy and prebiotics on the active fraction of the gut microbiota. Aids (2018) 32:1229–37. doi: 10.1097/QAD.0000000000001831 29683848

[19] Zhou Y Ou Z Tang X Zhou Y Xu H Wang X. Alterations in the gut microbiota of patients with acquired immune deficiency syndrome. J Cell Mol Med (2018) 22:2263–71. doi: 10.1111/jcmm.13508 PMC5867062 29411528

[20] Vujkovic-Cvijin I Dunham RM Iwai S Maher MC Albright RG Broadhurst MJ. Dysbiosis of the gut microbiota is associated with HIV disease progression and tryptophan catabolism. Sci Transl Med (2013) 5:(193):193ra91. doi: 10.1126/scitranslmed.3006438 PMC4094294 23843452

[21] Gori A Tincati C Rizzardini G Torti C Quirino T Haarman M. Early impairment of gut function and gut flora supporting a role for alteration of gastrointestinal mucosa in human immunodeficiency virus pathogenesis. J Clin Microbiol (2008) 46:757–8. doi: 10.1128/JCM.01729-07 PMC2238068 18094140

[22] Dinh DM Volpe GE Duffalo C Bhalchandra S Tai AK Kane AV. Intestinal microbiota, microbial translocation, and systemic inflammation in chronic HIV infection. J Infect Dis (2015) 211:19–27. doi: 10.1093/infdis/jiu409 25057045 PMC4326316

[23] Taylor MW Feng G. Relationship between interferon-γ, indoleamine 2,3-dioxygenase, and tryptophan catabolism. FASEB J (1991) 5:2516–22. doi: 10.1096/fasebj.5.11.1907934 1907934

[24] Wang Z Qi Q. Gut microbial metabolites associated with HIV infection. Future Virol (2019) 14:335–47. doi: 10.2217/fvl-2019-0002 PMC6595475 31263508

[25] Sui Y Dzutsev A Venzon D Frey B Thovarai V Trinchieri G. Influence of gut microbiome on mucosal immune activation and SHIV viral transmission in naive macaques article. Mucosal Immunol (2018) 11:1219–29. doi: 10.1038/s41385-018-0029-0 PMC6030500 29858581

[26] Moeller AH Shilts M Li Y Rudicell RS Lonsdorf EV Pusey AE. Siv-induced instability of the chimpanzee gut microbiome. Cell Host Microbe (2013) 14:340–5. doi: 10.1016/j.chom.2013.08.005 PMC3802538 24034619

[27] Ling Z Jin C Xie T Cheng Y Li L Wu N. Alterations in the fecal microbiota of patients with HIV-1 infection: An observational study in a Chinese population. Sci Rep (2016) 6:30673. doi: 10.1038/srep30673 27477587 PMC4967929

[28] González-Hernández LA Ruiz-Briseño MDR Sánchez-Reyes K Alvarez-Zavala M Vega-Magaña N López-Iñiguez A. Alterations in bacterial communities, SCFA and biomarkers in an elderly HIV-positive and HIV-negative population in western Mexico. BMC Infect Dis (2019) 19:1–12. doi: 10.1186/s12879-019-3867-9 30845929 PMC6407185

[29] Turnbaugh PJ Ley RE Mahowald MA Magrini V Mardis ER Gordon JI. An obesity-associated gut microbiome with increased capacity for energy harvest. Nature (2006) 444:1027–31. doi: 10.1038/nature05414 17183312

[30] Rajilić-Stojanović M Biagi E Heilig HGHJ Kajander K Kekkonen RA Tims S. Global and deep molecular analysis of microbiota signatures in fecal samples from patients with irritable bowel syndrome. Gastroenterology (2011) 141:1792–801. doi: 10.1053/j.gastro.2011.07.043 21820992

[31] Ling Z Liu X Jia X Cheng Y Luo Y Yuan L. Impacts of infection with different toxigenic clostridium difficile strains on faecal microbiota in children. Sci Rep (2014) 4:7485. doi: 10.1038/srep07485 25501371 PMC4265774

[32] Mariat D Firmesse O Levenez F Guimarǎes VD Sokol H Doré J. The firmicutes/bacteroidetes ratio of the human microbiota changes with age. BMC Microbiol (2009) 9:123. doi: 10.1186/1471-2180-9-123 19508720 PMC2702274

[33] Chen Y Lin H Cole M Morris A Martinson J Mckay H. Signature changes in gut microbiome are associated with increased susceptibility to HIV-1 infection in MSM. Microbiome (2021) 9:1–18. doi: 10.1186/s40168-021-01168-w 34879869 PMC8656045

[34] Russo E Nannini G Sterrantino G Kiros ST Di Pilato V Coppi M. Effects of viremia and CD4 recovery on gut “microbiome-immunity” axis in treatment-naïve HIV-1-infected patients undergoing antiretroviral therapy. World J Gastroenterol (2022) 28:635–52. doi: 10.3748/wjg.v28.i6.635 PMC8900548 35317423

[35] Vujkovic-Cvijin I Sortino O Verheij E Sklar J Wit FW Kootstra NA. HIV-Associated gut dysbiosis is independent of sexual practice and correlates with noncommunicable diseases. Nat Commun (2020) 11(1):2448. doi: 10.1038/s41467-020-16222-8 32415070 PMC7228978

[36] Williams B Weber K Chlipala G Evans C Morack R French A. HIV Status does not affect rectal microbiome composition, diversity, or stability over time: A Chicago women’s interagency HIV study. AIDS Res Hum Retroviruses (2019) 35:260–6. doi: 10.1089/aid.2018.0250 PMC6434590 30618262

[37] Wang Z Usyk M Sollecito CC Qiu Y Williams-Nguyen J Hua S. Altered gut microbiota and host metabolite profiles in women with human immunodeficiency virus. Clin Infect Dis (2020) 71:2345–53. doi: 10.1093/cid/ciz1117 PMC7713676 31748797

[38] Flygel TT Sovershaeva E Claassen-Weitz S Hjerde E Mwaikono KS Odland J. Composition of gut microbiota of children and adolescents with perinatal human immunodeficiency virus infection taking antiretroviral therapy in Zimbabwe. J Infect Dis (2020) 221:483–92. doi: 10.1093/infdis/jiz473 PMC7457326 31549151

[39] Sessa L Reddel S Manno E Quagliariello A Cotugno N Del Chierico F. Distinct gut microbiota profile in antiretroviral therapy-treated perinatally HIV-infected patients associated with cardiac and inflammatory biomarkers. Aids (2019) 33:1001–11. doi: 10.1097/QAD.0000000000002131 30946154

[40] Kaur US Shet A Rajnala N Gopalan BP Moar P Himanshu D. High abundance of genus prevotella in the gut of perinatally HIV-infected children is associated with IP-10 levels despite therapy. Sci Rep (2018) 8:1–16. doi: 10.1038/s41598-018-35877-4 30518941 PMC6281660

[41] Dieffenbach CW Fauci AS. Thirty years of HIV and AIDS: Future challenges and opportunities. Ann Intern Med (2011) 154:766–71. doi: 10.7326/0003-4819-154-11-201106070-00345 21628350

[42] Li SX Armstrong AJS Neff CP Shaffer M Lozupone CA Palmer BE. Complexities of gut microbiome dysbiosis in the context of HIV infection and antiretroviral therapy. Clin Pharmacol Ther (2016) 99:600–11. doi: 10.1002/cpt.363 PMC4927263 26940481

[43] Kartalija M Sande MA. Diarrhea and AIDS in the era of highly active antiretroviral therapy. Clin Infect Dis (1999) 28:701–7. doi: 10.1086/515191 10825021

[44] De Carvalho Santos ASA Silveira EA De Oliveira Falco M. Gastrointestinal symptoms in HIV-infected patients: Female sex and smoking as risk factors in an outpatient cohort in Brazil. PloS One (2016) 11(10):e0164774. doi: 10.1371/journal.pone.0164774 27749931 PMC5066957

[45] Mutlu EA Keshavarzian A Losurdo J Swanson G Siewe B Forsyth C. A compositional look at the human gastrointestinal microbiome and immune activation parameters in HIV infected subjects. PloS Pathog (2014) 10(2):e1003829. doi: 10.1371/journal.ppat.1003829 24586144 PMC3930561

[46] McHardy IH Li X Tong M Ruegger P Jacobs J Borneman J. HIV Infection is associated with compositional and functional shifts in the rectal mucosal microbiota. Microbiome (2013) 1:1. doi: 10.1186/2049-2618-1-26 24451087 PMC3971626

[47] Pérez-Santiago J Gianella S Massanella M Spina CA Karris MY Var SR. Gut lactobacillales are associated with higher CD4 and less microbial translocation during HIV infection. Aids (2013) 27:1921–31. doi: 10.1097/QAD.0b013e3283611816 PMC3816380 24180001

[48] Lozupone CA Li M Campbell TB Flores SC Linderman D Gebert MJ. Alterations in the gut microbiota associated with HIV-1 infection. Cell Host Microbe (2013) 14:329–39. doi: 10.1016/j.chom.2013.08.006 PMC3864811 24034618

[49] Tincati C Douek DC Marchetti G. Gut barrier structure, mucosal immunity and intestinal microbiota in the pathogenesis and treatment of HIV infection. AIDS Res Ther (2016) 13:1–11. doi: 10.1186/s12981-016-0103-1 27073405 PMC4828806

[50] Siddiqui S Bao D Doyle-Meyers L Dufour J Wu Y Liu YZ. Alterations of the gut bacterial microbiota in rhesus macaques with SIV infection and on short- or long-term antiretroviral therapy. Sci Rep (2020) 10:1–13. doi: 10.1038/s41598-020-76145-8 33149234 PMC7642356

[51] Ellis CL Ma ZM Mann SK Li CS Wu J Knight TH. Molecular characterization of stool microbiota in hiv-infected subjects by panbacterial and order-level 16s ribosomal DNA (rDNA) quantification and correlations with immune activation. J Acquir Immune Defic Syndr (1988) (2011) 57:363–70. doi: 10.1097/QAI.0b013e31821a603c PMC3153564 21436711

[52] Willing BP Dicksved J Halfvarson J Andersson AF Lucio M Zheng Z. A pyrosequencing study in twins shows that gastrointestinal microbial profiles vary with inflammatory bowel disease phenotypes. Gastroenterology (2010) 139:1844–1854.e1. doi: 10.1053/j.gastro.2010.08.049 20816835

[53] Krashin DL Merrill JO Trescot AM. Opioids in the management of HIV-related pain. Pain Physician (2012) 15:157–68. doi: 10.36076/ppj.2012/15/es157 22786454

[54] Lee K Vuong HE Nusbaum DJ Hsiao EY Evans CJ Taylor AMW. The gut microbiota mediates reward and sensory responses associated with regimen-selective morphine dependence. Neuropsychopharmacology (2018) 43:2606–14. doi: 10.1038/s41386-018-0211-9 PMC6224506 30258112

[55] Simpson S Mclellan R Wellmeyer E Matalon F George O. Drugs and bugs: the gut-brain axis and substance use disorders. J Neuroimmune Pharmacol (2022) 17:33–61. doi: 10.1007/s11481-021-10022-7 PMC9074906 34694571

[56] Akbarali HI Dewey WL. Gastrointestinal motility, dysbiosis and opioid-induced tolerance: is there a link? Nat Rev Gastroenterol Hepatol (2019) 16:323–4. doi: 10.1038/s41575-019-0150-x PMC7045338 31024090

[57] Xu Y Xie Z Wang H Shen Z Guo Y Gao Y. Bacterial diversity of intestinal microbiota in patients with substance use disorders revealed by 16S rRNA gene deep sequencing. Sci Rep (2017) 7:1–9. doi: 10.1038/s41598-017-03706-9 28620208 PMC5472629

[58] Vincent C Miller MA Edens TJ Mehrotra S Dewar K Manges AR. Bloom and bust: Intestinal microbiota dynamics in response to hospital exposures and clostridium difficile colonization or infection. Microbiome (2016) 4:12. doi: 10.1186/s40168-016-0156-3 26975510 PMC4791782

[59] Barengolts E Green SJ Eisenberg Y Akbar A Reddivari B Layden BT. Gut microbiota varies by opioid use, circulating leptin and oxytocin in African American men with diabetes and high burden of chronic disease. PloS One (2018) 13(3):e0194171. doi: 10.1371/journal.pone.0194171 29596446 PMC5875756

[60] Smith JP Bingaman SI Ruggiero F Mauger DT Mukherjee A McGovern CO. Therapy with the opioid antagonist naltrexone promotes mucosal healing in active crohn’s disease: A randomized placebo-controlled trial. Dig Dis Sci (2011) 56:2088–97. doi: 10.1007/s10620-011-1653-7 PMC3381945 21380937

[61] Wang F Roy S. Gut homeostasis, microbial dysbiosis, and opioids. Toxicol Pathol (2017) 45:150–6. doi: 10.1177/0192623316679898 PMC8607522 27895265

[62] Mora AL Salazar M Pablo-Caeiro J Frost CP Yadav Y Dupont HL. Moderate to high use of opioid analgesics are associated with an increased risk of clostridium difficile infection. Am J Med Sci (2012) 343:277–80. doi: 10.1097/MAJ.0b013e31822f42eb 21934595

[63] Babrowski T Holbrook C Moss J Gottlieb L Valuckaite V Zaborin A. Pseudomonas aeruginosa virulence expression is directly activated by morphine and is capable of causing lethal gut-derived sepsis in mice during chronic morphine administration. Ann Surg (2012) 255:386–93. doi: 10.1097/SLA.0b013e3182331870 PMC3258463 21989372

[64] Meng J Banerjee S Li D Sindberg GM Wang F Ma J. Opioid exacerbation of gram-positive sepsis, induced by gut microbial modulation, is rescued by IL-17A neutralization. Sci Rep (2015) 5:1–17. doi: 10.1038/srep10918 PMC4454150 26039416

[65] Banerjee S Sindberg G Wang F Meng J Sharma U Zhang L. Opioid-induced gut microbial disruption and bile dysregulation leads to gut barrier compromise and sustained systemic inflammation. Mucosal Immunol (2016) 9:1418–28. doi: 10.1038/mi.2016.9 PMC4996771 26906406

[66] Kang M Mischel RA Bhave S Komla E Cho A Huang C. The effect of gut microbiome on tolerance to morphine mediated antinociception in mice. Sci Rep (2017) 7:42658. doi: 10.1038/srep42658 28211545 PMC5314392

[67] Acharya C Betrapally NS Gillevet PM Sterling RK Akbarali H White MB. Chronic opioid use is associated with altered gut microbiota and predicts readmissions in patients with cirrhosis. Aliment Pharmacol Ther (2017) 45:319–31. doi: 10.1111/apt.13858 27868217

[68] Zhang J Deji C Fan J Chang L Miao X Xiao Y. Differential alteration in gut microbiome profiles during acquisition, extinction and reinstatement of morphine-induced CPP. Prog Neuropsychopharmacol Biol Psychiatry (2021) 104:110058. doi: 10.1016/J.PNPBP.2020.110058 32791167

[69] Wang F Meng J Zhang L Johnson T Chen C Roy S. Morphine induces changes in the gut microbiome and metabolome in a morphine dependence model. Sci Rep (2018) 8:1–15. doi: 10.1038/s41598-018-21915-8 29483538 PMC5827657

[70] Gicquelais RE Bohnert ASB Thomas L Foxman B. Opioid agonist and antagonist use and the gut microbiota: associations among people in addiction treatment. Sci Rep (2020) 10:1–11. doi: 10.1038/s41598-020-76570-9 33173098 PMC7655955

[71] Touw K Ringus DL Hubert N Wang Y Leone VA Nadimpalli A. Mutual reinforcement of pathophysiological host-microbe interactions in intestinal stasis models. Physiol Rep (2017) 5(6):e13182. doi: 10.14814/phy2.13182 28320888 PMC5371559

[72] Vinolo MAR Rodrigues HG Nachbar RT Curi R. Regulation of inflammation by short chain fatty acids. Nutrients (2011) 3:858–76. doi: 10.3390/nu3100858 PMC3257741 22254083

[73] Connery HS. Medication-assisted treatment of opioid use disorder: Review of the evidence and future directions. Harv Rev Psychiatry (2015) 23:63–75. doi: 10.1097/HRP.0000000000000075 25747920

[74] Cruz-Lebrón A Johnson R Mazahery C Troyer Z Joussef-Piña S Quiñones-Mateu ME. Chronic opioid use modulates human enteric microbiota and intestinal barrier integrity. Gut Microbes (2021) 13(1):1946368. doi: 10.1080/19490976.2021.1946368 PMC8317955 34313547

[75] Chelakkot C Ghim J Ryu SH. Mechanisms regulating intestinal barrier integrity and its pathological implications. Exp Mol Med (2018) 50:1–9. doi: 10.1038/s12276-018-0126-x PMC6095905 30115904

[76] Chavis NS Klein PW Cohen SM Dempsey A Hauck H Cheever LW. The health resources and services administration (HRSA) ryan white HIV/AIDS program’s response to the opioid epidemic. J Infect Dis (2020) 222:S477–85. doi: 10.1093/infdis/jiaa230 32877537

[77] Johnson SD Fox HS Buch S Byrareddy SN. Chronic opioid administration is associated with prevotella-dominated dysbiosis in SIVmac251 infected, cART-treated macaques. J Neuroimmune Pharmacol (2022) 17:3–14. doi: 10.1007/s11481-021-09993-4 PMC9969301 33788119

[78] Sindberg GM Callen SE Banerjee S Meng J Hale VL Hegde R. Morphine potentiates dysbiotic microbial and metabolic shifts in acute SIV infection. J Neuroimmune Pharmacol (2019) 14:200–14. doi: 10.1007/s11481-018-9805-6 PMC6917429 30242614

[79] Meng J Banerjee S Zhang L Sindberg G Moidunny S Li B. Opioids impair intestinal epithelial repair in HIV-infected humanized mice. Front Immunol (2020) 10:2999. doi: 10.3389/fimmu.2019.02999 32010125 PMC6978907

[80] Chang PV Hao L Offermanns S Medzhitov R. The microbial metabolite butyrate regulates intestinal macrophage function *via* histone deacetylase inhibition. Proc Natl Acad Sci U.S.A. (2014) 111:2247–52. doi: 10.1073/pnas.1322269111 PMC3926023 24390544

[81] Laserna-Mendieta EJ Clooney AG Carretero-Gomez JF Moran C Sheehan D Nolan JA. Determinants of reduced genetic capacity for butyrate synthesis by the gut microbiome in crohn’s disease and ulcerative colitis. J Crohns Colitis (2018) 12:204–16. doi: 10.1093/ecco-jcc/jjx137 29373727

[82] Ahmad MS Krishnan S Ramakrishna BS Mathan M Pulimood AB Murthy SN. Butyrate and glucose metabolism by colonocytes in experimental colitis in mice. Gut (2000) 46:493–9. doi: 10.1136/gut.46.4.493 PMC1727901 10716678

[83] Wang HB Wang PY Wang X Wan YL Liu YC. Butyrate enhances intestinal epithelial barrier function *via* up-regulation of tight junction protein claudin-1 transcription. Dig Dis Sci (2012) 57:3126–35. doi: 10.1007/s10620-012-2259-4 22684624

[84] Bouza E Rodriguez-Créixems M. Bacteremic infections in the HIV-infected patient and recurrent bacteremia. Clin Microbiol Infection (1999) 5:2s33–9. doi: 10.1111/j.1469-0691.1999.tb00540.x

[85] Zhang R Meng J Lian Q Chen X Bauman B Chu H. Prescription opioids are associated with higher mortality in patients diagnosed with sepsis: A retrospective cohort study using electronic health records. PloS One (2018) 13:1–8. doi: 10.1371/journal.pone.0190362 PMC5749778 29293575

[86] Oh NS Joung JY Lee JY Kim Y. Probiotic and anti-inflammatory potential of lactobacillus rhamnosus 4B15 and lactobacillus gasseri 4M13 isolated from infant feces. PloS One (2018) 13:1–15. doi: 10.1371/journal.pone.0192021 PMC5812581 29444150

[87] Iljazovic A Roy U Gálvez EJC Lesker TR Zhao B Gronow A. Perturbation of the gut microbiome by prevotella spp. enhances host susceptibility to mucosal inflammation. Mucosal Immunol (2021) 14:113–24. doi: 10.1038/s41385-020-0296-4 PMC7790746 32433514

[88] Maeda Y Takeda K. Host–microbiota interactions in rheumatoid arthritis. Exp Mol Med (2019) 51:1–6. doi: 10.1038/s12276-019-0283-6 PMC6906371 31827063

[89] Hatziioannou T Evans DT. Animal models for HIV/AIDS research. Nat Rev Microbiol (2012) 10:852–67. doi: 10.1038/nrmicro2911 PMC4334372 23154262

[90] Spanagel R. Animal models of addiction (2017). Available at: www.dialogues-cns.org. 10.31887/DCNS.2017.19.3/rspanagel PMC5741108 29302222

[91] Slivicki RA Earnest T Chang Y-H Pareta R Casey E Li JN. Oral oxycodone self-administration leads to features of opioid addiction in male and female mice. Addict Biol (2023) 28(1):e13253. doi: 10.1111/adb.13253 36577735 PMC11824864

[92] Irvine SL Hummelen R Hekmat S Looman C WN Habbema JDF Reid G. Probiotic yogurt consumption is associated with an increase of CD4 count among people living with HIV/AIDS. J Clin Gastroenterol (2010) 44:e201–5. doi: 10.1097/MCG.0b013e3181d8fba8 20463586

[93] Falasca K Vecchiet J Ucciferri C di Nicola M D’Angelo C Reale M. Effect of probiotic supplement on cytokine levels in HIV-infected individuals: A preliminary study. Nutrients (2015) 7:8335–47. doi: 10.3390/nu7105396 PMC4632416 26426044

[94] Zhang L Meng J Ban Y Jalodia R Chupikova I Fernandez I. Morphine tolerance is attenuated in germfree mice and reversed by probiotics, implicating the role of gut microbiome. Proc Natl Acad Sci U.S.A. (2019) 116:13523–32. doi: 10.1073/pnas.1901182116 PMC6613141 31209039

[95] Yang OO Kelesidis T Cordova R Khanlou H. Immunomodulation of antiretroviral drug-suppressed chronic HIV-1 infection in an oral probiotic double-blind placebo-controlled trial. AIDS Res Hum Retroviruses (2014) 30:988–95. doi: 10.1089/aid.2014.0181 PMC6461151 25127924

[96] Cunningham-Rundles S Ahrné S Johann-Liang R Abuav R Dunn-Navarra AM Grassey C. Effect of probiotic bacteria on microbial host defense, growth and immune function in human immunodeficiency virus type-1 infection. Nutrients (2011) 3:1042–70. doi: 10.3390/nu3121042 PMC3260491 22292110

[97] D’Ettorre G Ceccarelli G Giustini N Serafino S Calantone N De Girolamo G. Probiotics reduce inflammation in antiretroviral treated, HIV-infected individuals: Results of the “Probio-HIV” clinical trial. PloS One (2015) 10:1–15. doi: 10.1371/journal.pone.0137200 PMC4573418 26376436

[98] Gori A Rizzardini G Van’T Land B Amor KB Van Schaik J Torti C. Specific prebiotics modulate gut microbiota and immune activation in HAART-naive HIV-infected adults: Results of the “cOPA” pilot randomized trial. Mucosal Immunol (2011) 4:554–63. doi: 10.1038/mi.2011.15 PMC3158294 21525866

[99] Malazogu F Rousseau RK Shivappa N Huibner S Walmsley SL Kovacs CM. The dietary inflammatory index is not associated with gut permeability or biomarkers of systemic inflammation in HIV immunologic non-responders. Front Nutr (2021) 8:736816. doi: 10.3389/fnut.2021.736816 34881278 PMC8646029

[100] Rosel-Pech C Chávez-Torres M Bekker-Méndez VC Pinto-Cardoso S. Therapeutic avenues for restoring the gut microbiome in HIV infection. Curr Opin Pharmacol (2020) 54:188–201. doi: 10.1016/j.coph.2020.09.010 33271427

[101] Vujkovic-Cvijin I Rutishauser RL Pao M Hunt PW Lynch SV McCune JM. Limited engraftment of donor microbiome *via* one-time fecal microbial transplantation in treated HIV-infected individuals. Gut Microbes (2017) 8:440–50. doi: 10.1080/19490976.2017.1334034 PMC5628639 28541799

[102] Serrano-Villar S Talavera-Rodríguez A Gosalbes MJ Madrid N Pérez-Molina JA Elliott RJ. Fecal microbiota transplantation in HIV: A pilot placebo-controlled study. Nat Commun (2021) 12:1–13. doi: 10.1038/s41467-021-21472-1 33602945 PMC7892558

[103] Hensley-McBain T Zevin AS Manuzak J Smith E Gile J Miller C. Effects of fecal microbial transplantation on microbiome and immunity in simian immunodeficiency virus-infected macaques. J Virol (2016) 90:4981–9. doi: 10.1128/jvi.00099-16 PMC4859719 26937040

[104] Weingarden A González A Vázquez-Baeza Y Weiss S Humphry G Berg-Lyons D. Dynamic changes in short- and long-term bacterial composition following fecal microbiota transplantation for recurrent clostridium difficile infection. Microbiome (2015) 3:1–8. doi: 10.1186/s40168-015-0070-0 25825673 PMC4378022

[105] Gathe JC Diejomaoh EM Mayberry CC Clemmons JB. Fecal transplantation for clostridium difficile - “all stool may not be created equal”. J Int Assoc Provid AIDS Care (2016) 15:107–8. doi: 10.1177/2325957415627695 26821578

[106] Martínez-Ayala P González-Hernández LA Amador-Lara F Andrade-Villanueva J Ramos-Solano M. Fecal microbiota transplantation for severe complicated c. difficile colitis in a patient with acquired immunodeficiency syndrome. Rev Gastroenterología México (English Edition) (2019) 84:110–2. doi: 10.1016/j.rgmxen.2018.03.008 29449093

[107] Hakimian JK Dong TS Barahona JA Lagishetty V Tiwari S Azani D. Dietary supplementation with omega-3 polyunsaturated fatty acids reduces opioid-seeking behaviors and alters the gut microbiome. Nutrients (2019) 11(8):1900. doi: 10.3390/NU11081900 31416242 PMC6723154

[108] Buydens-Branchey L Branchey M. N-3 polyunsaturated fatty acids decrease anxiety feelings in a population of substance abusers. J Clin Psychopharmacol (2006) 26:661–5. doi: 10.1097/01.jcp.0000246214.49271.f1 17110827

[109] Joffre C Rey C Layé S. N-3 polyunsaturated fatty acids and the resolution of neuroinflammation. Front Pharmacol (2019) 10:1022. doi: 10.3389/fphar.2019.01022 31607902 PMC6755339

[110] Smith JP Book SW. Anxiety and substance use disorders: a review. Psychiatr Times (2008) 25(10):19–23. 20640182 PMC2904966

[111] Thomaz AC Iyer V Woodward TJ Hohmann AG. Fecal microbiota transplantation and antibiotic treatment attenuate naloxone-precipitated opioid withdrawal in morphine-dependent mice. Exp Neurol (2021) 343:113787. doi: 10.1016/j.expneurol.2021.113787 34153321 PMC8477666

